# Is the United States Still Dominant in the Global Pharmaceutical Innovation Network?

**DOI:** 10.1371/journal.pone.0077247

**Published:** 2013-11-05

**Authors:** Yuanjia Hu, Thomas Scherngell, Si Nga Man, Yitao Wang

**Affiliations:** 1 State Key Laboratory of Quality Research in Chinese Medicine, Institute of Chinese Medical Sciences, University of Macau, Taipa, Macao, China; 2 Foresight & Policy Development Department, AIT Austrian Institute of Technology, Vienna, Austria; Université de Montréal, Canada

## Abstract

The dramatic growth of research and development activities in the pharmaceutical sector in emerging economies raises the question of whether the United States still keeps its dominant role in the global pharmaceutical innovation landscape. This paper focuses on investigating the role of the United States in global pharmaceutical innovation, and differs from previous studies by shifting attention to a network analytic perspective to track the global distribution of pharmaceutical inventions. Our sample is composed of key patents covering all new drugs approved by the Food and Drug Administration between 1996 and 2010. The results show that the United States still dominates in the global pharmaceutical innovation network, especially when it comes to essential core inventions. However, the United States shows a slightly decreasing prominence in the networks of either total new drugs or New Molecular Entity (NME) drugs in the time period 2006–2010 as compared to previous time periods, revealed by subtle traces of network centralities.

## Introduction

Today it is widely agreed and empirically documented that the United States (US) has dominated global pharmaceutical innovation over the past decades. The US pharmaceutical sector is characterized by an extensive Research and Development (R&D) infrastructure and a comprehensive talent pool, an appropriate scientific regulatory system, and an advanced environment for fostering investments in new drug discovery and development. However, different recent developments may point to a decreasing tendency in the dominance of the US in pharmaceutical R&D activity over the last five to ten years [1]. Especially, public R&D budgets continuously cut due to a relative shrinking economy facing fiscal-cliff [2]–[8]. Thus, the US has shown a negative growth in clinical trials which is the crucial link between laboratory bench and patient bedside and increasingly going global in recent years [9]–[11].

Furthermore, emerging countries are developing at an accelerated pace in the domain of science, technology and innovation in the pharmaceutical sector, recognized as an important engine for sustainable economic growth in general [12]. China may constitute the most prominent example in this context, that has meanwhile ascended to the second-largest sponsor of global R&D, whether measured in terms of funding or generation of intellectual capital [6]. Moreover, it is predicted that China will be the second largest pharmaceutical market after the US by 2015 [13] which is, among other reasons, related to the launch of the project “Key Drug Innovation” by the Chinese government in 2007. The programme provides R&D funding to the pharmaceutical sector with an amount of $1 billion during 2011–2015, and will possibly increase to about $4.3 billion by 2020 [14], [15].

While China is the most striking example, numerous other emerging economies, such as India or South Korea, increasingly invest in pharmaceutical R&D. This raises the crucial question whether the US still can keep its dominant role in the global pharmaceutical innovation landscape.; Losing leadership in pharmaceutical innovation may result in a decreasing ability to capture value from new pharmaceutical products and devices, and, potentially more importantly, a decreasing ability to influence technological pathways and trajectories.

Recent works seem to confirm the dominant status of the US in the global pharmaceutical landscape by investigating the geographical location of pharmaceutical innovation, as captured by patent applications referring to new drugs [16]. Friedman explores the geographical location of pharmaceutical innovation during the time period 2000–2009 at the country level, focusing on the separate frequency of drug patent inventors of specific countries [16]. However, Friedman and related works do not consider cross-country interaction relationships in just separately examining the innovation capabilities for new drugs of specific countries. However, having in mind that research collaborations and networks are nowadays considered as essential elements to generate innovations in the knowledge based economy in general [17]–[19], and also in the pharmaceutical sector, the analysis of such cross-country R&D collaborations constitutes an important additional element when investigating the dominance of the US in global pharmaceutical innovation. This is related to the increasing complexity of finding new drugs, on the one hand, and, on the other hand, to the dramatically improving accessibility and mobility of research resources across geographical space [20].

Thus, we propose – as a complementary analysis to the country-centric view provided by [16] – a network analytic perspective to evaluate whether the US is still dominant in global pharmaceutical innovation. Such a perspective is useful for identifying and describing the structure of cross-country R&D collaboration flows – captured by cross-country co-patenting – in pharmaceutical industries, and also to capture the role and position of single countries in this network. This distinctive structural-relational emphasis sets our approach apart from individualistic, variable-centric traditions in the past. The main underlying assumption – coming from the literature on Social Networks [21] – in this context is that structural relations are often more important for understanding observed behaviours and resulting structures than are attributes of the actors [22]. In this sense, the network analytic perspective provides an important complementary picture to established exploratory and explanatory approaches, such as econometric modelling.

This paper focuses on investigating the role of the US in global pharmaceutical innovation, and differs from previous studies by shifting attention to a network analytic perspective to track the global distribution of pharmaceutical inventions. By this, we contribute in an innovative manner to the debate on whether the dominant role of the US in global pharmaceutical innovation decreases. Our approach is applicable for future assessments to understand the positioning of the US in global pharmaceutical innovation landscape.

## Methods

In order to identify the global distribution of pharmaceutical inventions, the first step is to determine how to measure the location of innovations, that is to say, which geographic indicator of innovations should be used. In the real world of pharmaceutical R&D, a lot of new drugs are developed based on collaborations between research laboratories of the same firms, especially multinational companies, or with universities located in the same clusters as either research laboratories or headquarters of firms. In this context, institutional-level indicators, for example, addresses of company headquarters or research laboratories, seem to be more informative to measure geographic distribution of inventions. However, it is extremely complicate to achieve standardized information of this kind of institutional-level indicators because of the evolution and restructuring of firms as well as relatively vague ownership relationships between inventions and research laboratories. However, individual level indicators based on patent inventors represent a promising alternative, avoiding the problems as above and able to precisely trace the origin of knowledge production.

In this article, we follow Friedman in using US patents to track the global distribution of R&D in the pharmaceutical sector [16]. We use such patents to construct our original dataset for the network analysis, based on the argument that the US is the most important worldwide pharmaceutical market, and, thus, act as benchmark in the scientific drug regulatory system. As we know, the US is the biggest pharmaceutical market in the world. Its market capacity reached $300 billion in 2009, comprising 40% of the world's pharmaceutical market [16]. Moreover, since its relatively rigorous regulatory system of drugs and Intellectual Property (IP), the US is always chosen as the first target market by the majority of worldwide pharmaceutical firms. It provides a good environment to attach to the global drug innovation community, and to enhance capabilities to generate new drug approvals in the US. In addition, patents are widely used as indicator to measure innovation, since patents do not only represent inventive outputs, but are closely associated with commercial application [23].

Our sample is composed of key patents covering all new drugs approved by the Food and Drug Administration (FDA) between 1996 and 2010. Key patents of a new drug are identified as relevant patents listed in the annual Orange Book in the same year to approve this drug, in order to avoid noise from insignificant and supplemental technology improvement at the late stage of pharmaceutical lifecycle. In this sense, the innovation in this study is exactly confined to be innovation outputs which are measured by patents related to marketed drugs, rather than innovation inputs or common innovation outputs, e.g. “sleeping” patents without contributions to real-world pharmaceutical products.

Totally, our dataset comprises 1967 patents of 1645 drugs. Country codes of inventors of sampled patents are retrieved from the database of the United States Patent and Trademark Office (USPTO). The country-specific patent frequency is based on patent inventors; cross-country collaboration strength is calculated on the basis of co-inventorship linkages using the integer method [24].

This set of relations within and between countries as measured by co-inventorship over all patents of our data sample may be described as a network A familiar representation is obtained by letting *V* be a set of nodes representing countries participating in the global pharmaceutical network, and *E* be a set of edges where elements of *E* are unordered pairs of distinct nodes *v_i_*, *v_j_* representing a link in the form of co-inventorships between a pair {*v_i_*, *v_j_*}. The two sets together are called a simple graph *G*_1_ =  (*V*, *E*) where all pairs {*v_i_*, *v_j_*}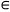 *E* are distinct and {*v_i_*, *v_i_*}∉ *E* for 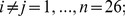 the number of edges incident on a vertex *i* = 1, …, *n* is called the degree *k_i_*. A path is the alternating sequence of vertices and links, beginning and ending with a vertex, so that the shortest path or geodesic distance *d_ij_* between two nodes *i* and *j* is defined as the number of vertices to be passed in the shortest possible path from one vertex to another.

Note that *G*_1_ represents an unweighted graph by definition. In our case, it is natural to consider the weighted form given by *G*_2_ =  (*V*, *E, W*) where *W* =  {*w*_1_, *w*_2_, …, *w_n_*} represent weights between two nodes *v_i_* and *v_j_* denoting the magnitude of co-inventorships between two countries. The topology of the graph is encoded in the *n×n* adjacency matrix **X** with elements
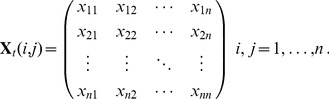
(1)


The SNA concept of centrality is a useful graph-theoretic approach for the purpose of this study. We focus on four different types of centrality measures [25], [26] that are calculated for each country in order to examine different aspects of network position: *First*, *degree centrality* is defined as the ratio of the degree of a node and the maximum degree in a network of the same size (i.e., the total number of edges connected to a node); Formally it is defined as
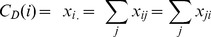
(2)so that it may be simply interpreted as the degree of prestige a node has due its simple number of connections to other nodes.

*Second*, *betweenness centrality* of a vertex can be defined as the fraction of geodesic paths between any pair of vertices on which this vertex lies. It is measured by the frequency of one actor positioned on the shortest path between other groups of actors arranged in pairs, given by
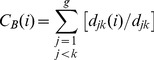
(3)where 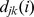 represents the shortest path between organisations *j* and *k* going through organisation *i*. Those actors, who are located on the shortest paths between many actors, therefore hold a key position for controlling the flow of information within the network (gatekeeper function).

*Third*, *closeness centrality* of a vertex is defined as the inverse of the mean geodesic distance (i.e., the mean length of the shortest path) from this vertex to every other vertex in a connected graph so that
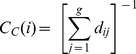
(4)


By this, it represents a measure of how close a node is located to all other nodes in the network by means of network structural characteristics, and, by this, how fast the node may get access to dispersed information in the network.

*Fourth*, *Eigenvector centrality* accords each vertex a centrality that depends both on the number and the quality of its connections by examining all vertices in parallel and assigning centrality weights that correspond to the average centrality of all neighbours; it is formally given by
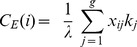
(5)where *λ* is the largest eigenvalue of **X**. A high Eigenvector centrality of a node indicates that this node is connected with other nodes that also show many connections, rather than to peripheral nodes.

## Results

**Figure 1:**
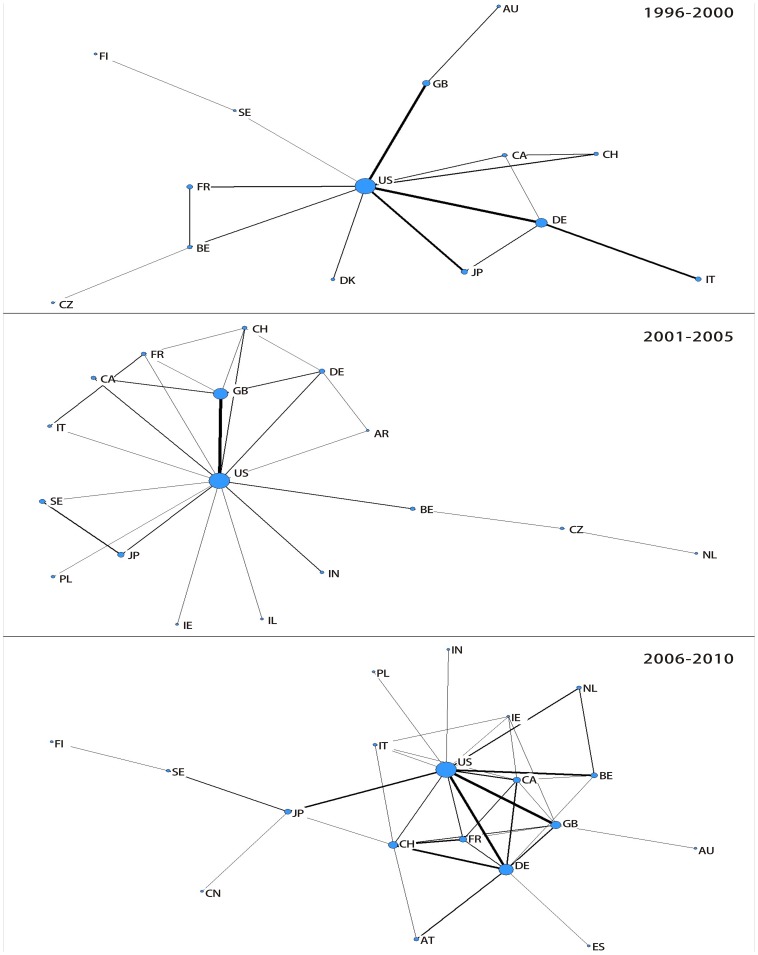
Global pharmaceutical innovation network. Note: AR Argentina, AT Austria, AU Australia, BE Belgium, CA Canada, CH Switzerland, CN China, CZ Czech Republic, DE Germany, DK Denmark, ES Spain, FI Finland, FR France, GB United Kingdom, IE Ireland, IL Israel, IN India, IT Italy, JP Japan, NL Netherlands, PL Poland, SE Sweden, US United States. Note: Vertex positions determined using spectral graph analytic methods according to the normalized Laplacian so that countries that are strongly interconnected positioned nearer to each other [27]. Node size corresponds to the weighted degree centrality of a country that is defined as the sum of a countrýs co-intventorships, the strength of the lines correspond to total co-inventorships between two countries.

In a first step, Figure 1 visualizes global networks of all new drugs using *G*_2_ for the time periods 1996–2000, 2001–2005 and 2006–2010 by means of information-theoretic techniques. We determine the position for the nodes (countries) using a standard approach from spectral graph analysis according to the normalized Laplacian, so that countries that show a relatively higher intensity of co-inventorships are positioned nearer to each other (for details see the discussion of the normalized graph Laplacian, in e.g. Higham and Kibble 2004) [27]. The node size corresponds to the weighted degree centrality of a country that is defined as the sum of a countrýs number of co-inventorships.

It can be seen clearly that the US represents the central hub in all time periods showing the highest interaction intensity with other countries. The most important partners in terms of absolute size are Germany and the United Kingdom for the time periods 1996–2000, the United Kingdom for the time period 2001–2005, and the United Kingdom, Germany as well as Belgium for the most recent time period 2006–2010. However, the most interesting results concerns the changing overall structure of the global pharmaceutical network of the observed three time periods. In general, interaction intensity increases over time. However, while this increased interaction is mostly subject to interactions with the US for the time period 2001–2005 (note that for this time period the network visualisation come close to a so-called star graph, where one hub in the centre connects all other nodes), interaction patterns becomes far more dispersed across different countries for the most recent time period, in particular due to a denser network structure among European countries that are located near to each other in geographical space. Furthermore, the entry of large emerging economies in the network in the most recent time period is observable. India enters directly connected via he US, while China becomes connected to the network via Japan.

Regarding absolute values of network centralities during 2006–2010, the US shows by far the highest value for all centrality measures that are taken into account, and, this, can be still considered as the dominant locus of drug innovation in the time periods under consideration. It can further be shown from the observed network centralities during 2006–2010 that different countries are on the second rank across different measures of centrality. Germany, for instance, shows a higher degree centrality in comparison to the United Kingdom, but a slightly higher Eigenvector centrality which is related to the fact that Germany has lower interaction intensity to the US than the United Kingdom.

In addition, it is necessary to discuss internal collaborations within a country because a lot of new drugs are developed based on this kind of collaborations. Note that the diagonal value of our network matrix represents the frequency of intra-country collaborations. As a result of the network for all new drugs during 2006–2010, the US, Germany and Japan are top 3 counties with the highest amount of regional internal connections, accounting for 52.38%, 13.04%, and 13.01% of total frequency, respectively. Hence, the US seems to be absolutely leading in terms of intra-country collaborations in the pharmaceutical sector.

However, the question that may be raised is whether these patterns can also be observed for different types of drugs. The original data actually cover various heterogeneous types of drugs, such as, New Molecular Entity (NME), new dosage form, new indication, and new combination. As is well known, NME generally represents the original core technology of innovative pharmaceuticals, which could be subsequently developed to other diversified derivative and complementary new drugs [28]–[30]. It is, thus, of great significance to analyse the role of US in global network by refining our original data sample into a new dataset containing NME drugs only, and further compare the differences between the two datasets, in order to understand the global landscape of pharmaceutical essential core inventions.

As a result, the US is still listed to be in the 1^st^ place in the network in terms of all centrality measures based on the dataset using NME drugs only. The comparative results on the different datasets are shown in Table 1. The US shows higher centralities for degree-, betweenness-, and closeness centrality, while only a slightly lower value for eigenvector centrality. That is to say, when only considering core inventions, the US is even more dominant in terms of degree-, betweenness-, and closeness centrality as compared to common technologies.

**Table 1:** Centrality percentage of countries in global drug innovation network: Total new drugs vs. NME drugs (2006–2010).

	US	Japan	Europe	Rest of the world
**Degree**				
** Total new drugs**	16.44%	5.48%	64.38%	13.70%
** NME**	22.50%	5.00%	60.00%	12.50%
** Difference**	6.06%	−0.48%	−4.38%	−1.20%
**Betweenness**				
** Total new drugs**	35.53%	21.96%	39.92%	2.59%
** NME**	55.02%	13.25%	31.73%	0.00%
** Difference**	19.49%	−8.71%	−8.19%	−2.59%
**Closeness**				
** Total new drugs**	5.01%	4.81%	65.40%	24.78%
** NME**	5.07%	4.95%	61.52%	28.46%
** Difference**	0.06%	0.14%	−3.88%	3.68%
**Eigenvector**				
** Total new drugs**	86.15%	2.04%	10.62%	1.19%
** NME**	80.37%	5.30%	12.55%	1.78%
** Difference**	−5.78%	3.26%	1.93%	0.59%

Note: The percentages in the rows of Difference are equal to values of relative NME minus values of according total new drugs. The differences are used to measure changes of the centrality share of countries from innovation network based on total new drugs to the network constructed by NME. The percentages in the total new drugs and NME refer to the share of national or regional centrality in total sum of relative centrality.

Finally, a longitudinal analysis is performed by using networks of total new drugs and NME drugs and respective centrality indicators with the aim of investigating the evolution of the US positioning in the global network over different time periods. In doing so, we compare three five-year periods, that is 1996–2000, 2001–2005, and 2006–2010. It can be clearly seen from Table 2 that the dominance of the US is significantly decreasing – though the US is still dominant as shown above – in the most recent time period 2006–2010 not only for total new drugs but NME drugs, in particular for degree centrality, betweenness centrality, and eigenvector centrality. This may be resulting from more frequent interaction relationships in general leading to a denser network structure among other countries than the US.

**Table 2:** The centrality share of the US in global drug innovation network.

Drug coverage	Periods	Degree	Betweenness	Closeness	Eigenvector
**Total new drugs**	1996–2000	26.47%	56.96%	5.11%	94.50%
	2001–2005	28.00%	68.76%	5.11%	90.77%
	2006–2010	16.44%	35.53%	5.01%	86.15%
**NME**	1996–2000	29.17%	63.83%	4.90%	88.11%
	2001–2005	24.00%	60.42%	4.95%	88.13%
	2006–2010	22.50%	55.02%	5.07%	80.37%

Note: The percentages in the cell refer to the share of the centrality of the US in total sum of relative centrality of all countries in global innovation network of specific drug coverage during specific time periods.

## Discussion

This study offers a network analytic approach to evaluate the dominance of the US in global pharmaceutical innovation. The results show that the US still dominates in the global pharmaceutical innovation network in terms of a visualized graph, absolute values of centrality measures, and regional internal innovation collaborations, especially when it comes to essential core inventions. However, it shows a slightly decreasing prominence in the networks of either total new drugs or NME drugs in the time period 2006–2010 as compared to previous time periods, revealed by subtle traces of network centralities.

This slight decrease in importance of the US in the network may be a reflection of falling shares of the US in the global GDP in general, and more importantly, in the worldwide pharmaceutical market. Between 1999 to 2009, the US share of worldwide R&D expenditure declined from 38% to 31% [4], and also, by 2015, the share of US in the global pharmaceutical market will fall to 31%, from a percentage of 41% in 2005 [31]. This is appropriate explanatory notes on the ground that the innovation is, to a certain extent, resulting from the investment which is considerably driven by the market [32], [33]. Furthermore, decreasing costs of technology may also play a role in the evloving innovation pattern. With decreasing costs of technology, especially concerning information technologies, it may become increasingly complicated for dominating actors to keep their predominant position due to a lower thresold of blocking followers; at the same time laggers are getting easier access to technology at lower costs. In addition, it is also possible that the focus of the pharmaceutical industry to move towards academia for partnerships somehow pushes collaborations with other countries over the US. However, all potential causes mentioned above, including R&D expenditures, pharmaceutical market developments, technology costs and science-industry partnerships need to be validated by further empirical research.

Before closing the article, let us return to the controversial issue on the development of pharmaceutical R&D in the US and emerging economies. So far, there exists a considerable number of indications pointing to a more prominent role of emerging countries in pharmaceutical R&D, in particular over the past decade. The important point to note is, however, that most of them concentrate on inputs into pharmaceutical R&D rather than on outputs. Only little measurable innovative output from emerging countries is observed by now, as also for R&D collaborations demonstrated by the network visualized in Figure 1, built on the basis of patents related to marketed drugs as key innovation outputs. Furthermore, the contradiction between innovation inputs and outputs in emerging countries could perhaps be explained by the considerable uncertainty of new drug discovery and the huge time lag between the initial discovery of potential drugs and market approvals. Generally speaking, bringing new drugs to the market has always been a rather risky and time-consuming activity with an average time frame of 10–15 years [34]. Therefore, the increasing importance of emerging economies in global drug innovation, and, thus, in the global pharmaceutical network, may become just visible in the near future when considering the R&D output. Thus, there is urgent need to continuously observe this network for future assessments to understand the positioning of the US in the global pharmaceutical innovation landscape.

Finally, some limitations of this research need to be noted, pointing to a future research agenda for analyzing global pharmaceutical networks. Firstly, the study uses key patents relevant to new drugs approved by the FDA as indicator to measure global pharmaceutical innovation. Though this kind of indicator fully considers inventions contributing to real-world pharmaceutical products and effectively avoids noise from insignificant and supplemental technology improvement at the late stage of pharmaceutical lifecycle, it cannot capture the considerable time lag of an invention to become productive, to a certain extent caused by long pharmaceutical R&D pipeline. Thus, it is necessary to regularly update current research results of the current study, and employing complementary innovation measurement indicators as, for example, scientific publications in order to examine the latest global pharmaceutical innovation pattern. Secondly, the research results are in the current research design simply described as a general profile of global drug innovation. It seems to be of great significance to conduct this analysis taking into account the therapeutic category of drug approved, and to identify the importance of the US in pharmaceutical innovation in specific therapeutic areas. Thirdly, extending the focus from pharmaceutical R&D to diagnostic and medical device or biotechnology innovation is – due to its closed and important relationship with pharmaceutical drugs – an important issue for future research.
